# Ethyl 4-(phenyl­sulfon­yl)piperazine-1-carboxyl­ate

**DOI:** 10.1107/S1600536811033162

**Published:** 2011-08-27

**Authors:** Mohammad T.M. Al-Dajani, Hassan H. Adballah, Nornisah Mohamed, Madhukar Hemamalini, Hoong-Kun Fun

**Affiliations:** a School of Pharmaceutical Sciences, Universiti Sains Malaysia, 11800 USM, Penang, Malaysia; b School of Chemical Sciences, Universiti Sains Malaysia, 11800 USM, Penang, Malaysia; c X-ray Crystallography Unit, School of Physics, Universiti Sains Malaysia, 11800 USM, Penang, Malaysia

## Abstract

In the title compound, C_13_H_18_N_2_O_4_S, the piperazine ring adopts a chair conformation. The dihedral angle between the least-squares planes through the piperazine and benzene rings is 73.23 (10)°. In the crystal, there are no classical hydrogen bonds but stabilization is provided by weak C—H⋯π inter­actions.

## Related literature

For the biological activity of piperazine derivatives, see: Emami *et al.* (2006 ▶); Foroumadi *et al.* (2007 ▶). For puckering parameters, see: Cremer & Pople (1975 ▶).
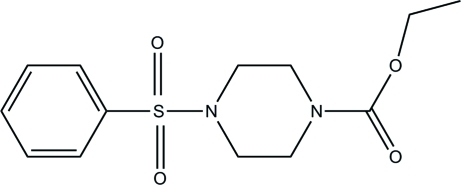


## Experimental

### 

#### Crystal data

C_13_H_18_N_2_O_4_S
*M* *_r_* = 298.35
Monoclinic, 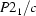

*a* = 6.1433 (5) Å
*b* = 20.5966 (17) Å
*c* = 12.5626 (8) Å
β = 114.026 (3)°
*V* = 1451.84 (19) Å^3^
*Z* = 4
Mo *K*α radiation
μ = 0.24 mm^−1^
*T* = 296 K
0.58 × 0.38 × 0.17 mm

#### Data collection

Bruker APEXII DUO CCD area-detector diffractometer
Absorption correction: multi-scan (*SADABS*; Bruker, 2009 ▶) *T*_min_ = 0.875, *T*_max_ = 0.961
16507 measured reflections
4255 independent reflections
3400 reflections with *I* > 2σ(*I*)
*R*_int_ = 0.023

#### Refinement

*R*[*F* ^2^ > 2σ(*F* ^2^)] = 0.048
*wR*(*F* ^2^) = 0.158
*S* = 1.04
4255 reflections
182 parameters
H-atom parameters constrained
Δρ_max_ = 0.60 e Å^−3^
Δρ_min_ = −0.30 e Å^−3^

### 

Data collection: *APEX2* (Bruker, 2009 ▶); cell refinement: *SAINT* (Bruker, 2009 ▶); data reduction: *SAINT*; program(s) used to solve structure: *SHELXTL* (Sheldrick, 2008 ▶); program(s) used to refine structure: *SHELXTL*; molecular graphics: *SHELXTL*; software used to prepare material for publication: *SHELXTL* and *PLATON* (Spek, 2009 ▶).

## Supplementary Material

Crystal structure: contains datablock(s) global, I. DOI: 10.1107/S1600536811033162/tk2781sup1.cif

Structure factors: contains datablock(s) I. DOI: 10.1107/S1600536811033162/tk2781Isup2.hkl

Supplementary material file. DOI: 10.1107/S1600536811033162/tk2781Isup3.cml

Additional supplementary materials:  crystallographic information; 3D view; checkCIF report

## Figures and Tables

**Table 1:** Hydrogen-bond geometry (Å, °) *Cg*1 is the centroid of the C1–C6 ring.

*D*—H⋯*A*	*D*—H	H⋯*A*	*D*⋯*A*	*D*—H⋯*A*
C13—H13*A*⋯*Cg*1^i^	0.96	2.97	3.900 (4)	165

Symmetry code: (i) 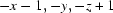.

**Fig. 1.**
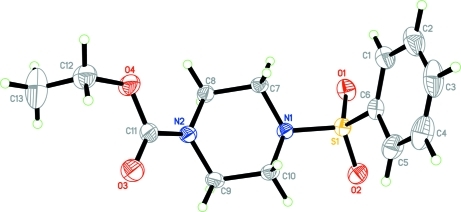
The molecular structure of (I), showing the atom labelling scheme and 50% probability displacement ellipsoids.

**Fig. 2.**
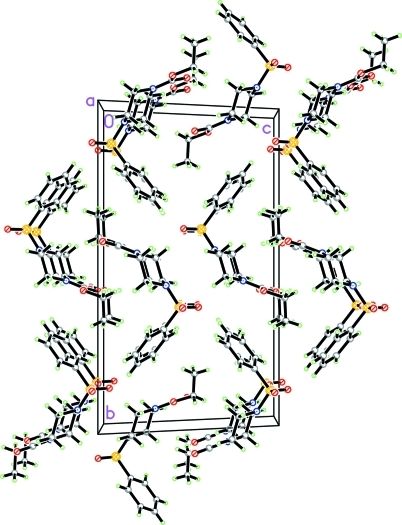
A view of the crystal packing in (I).

## supplementary crystallographic information

### Comment

Piperazine derivatives are used as antibiotic drugs, *e.g*. Norfloxacin, Ciprofloxacin, Enoxacin, Ofloxacin and Levofloxacine (Emami *et al.*, 2006; Foroumadi *et al.*, 2007). Due to the biological importance of piperazine, herein, we present the crystal and molecular structure of the title compound, (I).

The piperazine (N1–N2/C7–C10) ring in (I), Fig. 1, adopts a chair conformation [puckering parameters: Q = 0.5682 (18) Å, θ = 2.56 (17) ° and φ = 349 (4) ° (Cremer & Pople, 1975)] with atoms N1 and C9 deviating by 0.253 (1) and 0.223 (2) Å from the least-squares plane defined by the remaining atoms (N2/C7,C8/C10) in the ring. The dihedral angle between the piperazine (N1–N2/C7–C10) ring and the benzene (C1–C6) ring is 73.23 (10) °.

In the crystal structure (Fig. 2), there are no classical hydrogen bonds but stabilization is provided by weak C—H···π interactions (Table 1).

### Experimental

In a round bottom flask, 25ml of toluene was mixed with benzenesulfonyl chloride (0.01 mol, 1.0 g) with stirring. Ethyl-1-piperazine-carboxylate (0.01 mol, 1.7ml) dissolved in toluene was then added drop wise. The reaction mixture was refluxed for 30 min. The yellow precipitate formed was washed with alkaline water. The precipitate was then dissolved in methanol at room temperature. After few days, yellow crystals were formed by slow evaporation.

### Refinement

All hydrogen atoms were positioned geometrically [C–H = 0.93–0.97 Å] and were refined using a riding model, with *U*_iso_(H) = 1.2 or 1.5 *U*_eq_(C). A rotating group model was applied to the methyl group.

### Crystal data

**Table:**

C_13_H_18_N_2_O_4_S	*F*(000) = 632
*M**_r_* = 298.35	*D*_x_ = 1.365 Mg m^−^^3^
Monoclinic, *P*2_1_/*c*	Mo *K*α radiation, λ = 0.71073 Å
Hall symbol: -P 2ybc	Cell parameters from 5957 reflections
*a* = 6.1433 (5) Å	θ = 2.7–29.7°
*b* = 20.5966 (17) Å	µ = 0.24 mm^−^^1^
*c* = 12.5626 (8) Å	*T* = 296 K
β = 114.026 (3)°	Block, yellow
*V* = 1451.84 (19) Å^3^	0.58 × 0.38 × 0.17 mm
*Z* = 4	

### Data collection

**Table:**

Bruker APEXII DUO CCD area-detector diffractometer	4255 independent reflections
Radiation source: fine-focus sealed tube	3400 reflections with *I* > 2σ(*I*)
graphite	*R*_int_ = 0.023
φ and ω scans	θ_max_ = 30.1°, θ_min_ = 2.0°
Absorption correction: multi-scan (*SADABS*; Bruker, 2009)	*h* = −8→8
*T*_min_ = 0.875, *T*_max_ = 0.961	*k* = −29→24
16507 measured reflections	*l* = −17→17

### Refinement

**Table:**

Refinement on *F*^2^	Primary atom site location: structure-invariant direct methods
Least-squares matrix: full	Secondary atom site location: difference Fourier map
*R*[*F*^2^ > 2σ(*F*^2^)] = 0.048	Hydrogen site location: inferred from neighbouring sites
*wR*(*F*^2^) = 0.158	H-atom parameters constrained
*S* = 1.04	*w* = 1/[σ^2^(*F*_o_^2^) + (0.0887*P*)^2^ + 0.3986*P*] where *P* = (*F*_o_^2^ + 2*F*_c_^2^)/3
4255 reflections	(Δ/σ)_max_ = 0.001
182 parameters	Δρ_max_ = 0.60 e Å^−^^3^
0 restraints	Δρ_min_ = −0.30 e Å^−^^3^

### Special details

**Table:**

Geometry. All s.u.'s (except the s.u. in the dihedral angle between two l.s. planes) are estimated using the full covariance matrix. The cell s.u.'s are taken into account individually in the estimation of s.u.'s in distances, angles and torsion angles; correlations between s.u.'s in cell parameters are only used when they are defined by crystal symmetry. An approximate (isotropic) treatment of cell s.u.'s is used for estimating s.u.'s involving l.s. planes.
Refinement. Refinement of F^2^ against ALL reflections. The weighted R-factor wR and goodness of fit S are based on F^2^, conventional R-factors R are based on F, with F set to zero for negative F^2^. The threshold expression of F^2^ > 2σ(F^2^) is used only for calculating R-factors(gt) etc. and is not relevant to the choice of reflections for refinement. R-factors based on F^2^ are statistically about twice as large as those based on F, and R- factors based on ALL data will be even larger.

### Fractional atomic coordinates and isotropic or equivalent isotropic displacement parameters (Å^2^)

**Table:**

	*x*	*y*	*z*	*U*_iso_*/*U*_eq_	
S1	0.36299 (7)	−0.120155 (19)	0.94030 (3)	0.03858 (14)	
O1	0.3060 (3)	−0.12052 (6)	1.04000 (10)	0.0517 (3)	
O2	0.6074 (2)	−0.12032 (7)	0.95617 (12)	0.0547 (3)	
O3	−0.1090 (3)	0.07756 (8)	0.52229 (12)	0.0659 (4)	
O4	−0.3840 (2)	0.07205 (8)	0.59831 (12)	0.0605 (4)	
N1	0.2424 (2)	−0.05482 (6)	0.86538 (10)	0.0346 (3)	
N2	−0.0094 (2)	0.04239 (7)	0.70682 (12)	0.0412 (3)	
C1	0.0247 (4)	−0.21553 (9)	0.86231 (18)	0.0544 (4)	
H1A	−0.0323	−0.1999	0.9156	0.065*	
C2	−0.0839 (4)	−0.26783 (11)	0.7902 (2)	0.0749 (7)	
H2A	−0.2149	−0.2877	0.7957	0.090*	
C3	−0.0001 (5)	−0.29053 (11)	0.7108 (2)	0.0813 (8)	
H3A	−0.0749	−0.3255	0.6630	0.098*	
C4	0.1924 (5)	−0.26197 (12)	0.7021 (2)	0.0785 (7)	
H4A	0.2477	−0.2776	0.6482	0.094*	
C5	0.3063 (4)	−0.20992 (11)	0.77261 (18)	0.0595 (5)	
H5A	0.4375	−0.1905	0.7666	0.071*	
C6	0.2208 (3)	−0.18732 (8)	0.85247 (14)	0.0421 (3)	
C7	−0.0086 (3)	−0.04191 (8)	0.84341 (13)	0.0376 (3)	
H7A	−0.0340	−0.0501	0.9135	0.045*	
H7B	−0.1126	−0.0704	0.7824	0.045*	
C8	−0.0652 (3)	0.02844 (8)	0.80650 (14)	0.0406 (3)	
H8A	−0.2328	0.0367	0.7862	0.049*	
H8B	0.0271	0.0567	0.8708	0.049*	
C9	0.2364 (3)	0.02842 (9)	0.72652 (16)	0.0472 (4)	
H9A	0.3431	0.0565	0.7873	0.057*	
H9B	0.2594	0.0367	0.6558	0.057*	
C10	0.2931 (3)	−0.04207 (9)	0.76225 (14)	0.0423 (3)	
H10A	0.1969	−0.0703	0.6987	0.051*	
H10B	0.4597	−0.0508	0.7804	0.051*	
C11	−0.1625 (3)	0.06530 (8)	0.60275 (14)	0.0436 (4)	
C12	−0.5669 (4)	0.09306 (12)	0.48616 (18)	0.0637 (5)	
H12A	−0.5300	0.0763	0.4233	0.076*	
H12C	−0.7206	0.0759	0.4769	0.076*	
C13	−0.5775 (7)	0.16308 (16)	0.4804 (3)	0.1174 (13)	
H13A	−0.6971	0.1763	0.4064	0.176*	
H13B	−0.4254	0.1799	0.4891	0.176*	
H13C	−0.6170	0.1795	0.5418	0.176*	

### Atomic displacement parameters (Å^2^)

**Table:**

	*U*^11^	*U*^22^	*U*^33^	*U*^12^	*U*^13^	*U*^23^
S1	0.0397 (2)	0.0421 (2)	0.0286 (2)	0.00210 (13)	0.00853 (15)	0.00111 (13)
O1	0.0710 (9)	0.0526 (7)	0.0297 (6)	0.0009 (6)	0.0187 (6)	0.0025 (5)
O2	0.0368 (6)	0.0628 (8)	0.0521 (8)	0.0056 (5)	0.0053 (5)	0.0070 (6)
O3	0.0668 (9)	0.0926 (11)	0.0443 (7)	0.0176 (8)	0.0288 (6)	0.0217 (7)
O4	0.0449 (7)	0.0863 (10)	0.0481 (7)	0.0147 (6)	0.0167 (6)	0.0204 (7)
N1	0.0342 (6)	0.0394 (6)	0.0305 (6)	0.0018 (5)	0.0136 (5)	0.0023 (5)
N2	0.0382 (6)	0.0502 (7)	0.0376 (6)	0.0057 (5)	0.0179 (5)	0.0107 (6)
C1	0.0568 (10)	0.0465 (9)	0.0550 (10)	−0.0022 (8)	0.0178 (8)	0.0017 (8)
C2	0.0690 (14)	0.0462 (11)	0.0853 (17)	−0.0098 (9)	0.0065 (12)	0.0024 (11)
C3	0.0979 (19)	0.0399 (10)	0.0691 (15)	0.0061 (11)	−0.0039 (13)	−0.0143 (10)
C4	0.1010 (19)	0.0625 (14)	0.0608 (13)	0.0193 (13)	0.0213 (13)	−0.0212 (11)
C5	0.0699 (12)	0.0580 (11)	0.0521 (10)	0.0106 (9)	0.0262 (9)	−0.0092 (9)
C6	0.0470 (8)	0.0366 (8)	0.0374 (7)	0.0065 (6)	0.0119 (6)	0.0000 (6)
C7	0.0366 (7)	0.0439 (8)	0.0364 (7)	0.0003 (6)	0.0192 (6)	0.0037 (6)
C8	0.0435 (7)	0.0449 (8)	0.0373 (7)	0.0074 (6)	0.0205 (6)	0.0040 (6)
C9	0.0376 (7)	0.0587 (10)	0.0494 (9)	0.0020 (7)	0.0220 (7)	0.0164 (8)
C10	0.0380 (7)	0.0556 (9)	0.0386 (8)	0.0075 (6)	0.0210 (6)	0.0076 (7)
C11	0.0455 (8)	0.0477 (9)	0.0375 (8)	0.0038 (6)	0.0169 (6)	0.0053 (6)
C12	0.0510 (10)	0.0811 (15)	0.0494 (10)	0.0032 (10)	0.0104 (8)	0.0043 (10)
C13	0.131 (3)	0.077 (2)	0.101 (2)	0.0254 (18)	0.003 (2)	0.0136 (17)

### Geometric parameters (Å, °)

**Table:**

S1—O1	1.4312 (13)	C4—H4A	0.9300
S1—O2	1.4320 (14)	C5—C6	1.389 (2)
S1—N1	1.6365 (13)	C5—H5A	0.9300
S1—C6	1.7634 (17)	C7—C8	1.518 (2)
O3—C11	1.210 (2)	C7—H7A	0.9700
O4—C11	1.346 (2)	C7—H7B	0.9700
O4—C12	1.465 (2)	C8—H8A	0.9700
N1—C10	1.4745 (18)	C8—H8B	0.9700
N1—C7	1.4755 (18)	C9—C10	1.518 (2)
N2—C11	1.347 (2)	C9—H9A	0.9700
N2—C8	1.4554 (19)	C9—H9B	0.9700
N2—C9	1.455 (2)	C10—H10A	0.9700
C1—C6	1.387 (3)	C10—H10B	0.9700
C1—C2	1.391 (3)	C12—C13	1.444 (4)
C1—H1A	0.9300	C12—H12A	0.9700
C2—C3	1.377 (4)	C12—H12C	0.9700
C2—H2A	0.9300	C13—H13A	0.9600
C3—C4	1.365 (4)	C13—H13B	0.9600
C3—H3A	0.9300	C13—H13C	0.9600
C4—C5	1.385 (3)		
			
O1—S1—O2	119.62 (9)	C8—C7—H7B	109.9
O1—S1—N1	107.02 (7)	H7A—C7—H7B	108.3
O2—S1—N1	106.60 (7)	N2—C8—C7	110.26 (12)
O1—S1—C6	107.94 (8)	N2—C8—H8A	109.6
O2—S1—C6	108.04 (8)	C7—C8—H8A	109.6
N1—S1—C6	107.00 (7)	N2—C8—H8B	109.6
C11—O4—C12	115.91 (15)	C7—C8—H8B	109.6
C10—N1—C7	112.47 (11)	H8A—C8—H8B	108.1
C10—N1—S1	116.33 (10)	N2—C9—C10	109.69 (13)
C7—N1—S1	116.73 (10)	N2—C9—H9A	109.7
C11—N2—C8	126.01 (13)	C10—C9—H9A	109.7
C11—N2—C9	120.02 (13)	N2—C9—H9B	109.7
C8—N2—C9	113.96 (12)	C10—C9—H9B	109.7
C6—C1—C2	118.1 (2)	H9A—C9—H9B	108.2
C6—C1—H1A	121.0	N1—C10—C9	108.96 (13)
C2—C1—H1A	121.0	N1—C10—H10A	109.9
C3—C2—C1	120.9 (2)	C9—C10—H10A	109.9
C3—C2—H2A	119.6	N1—C10—H10B	109.9
C1—C2—H2A	119.6	C9—C10—H10B	109.9
C4—C3—C2	120.2 (2)	H10A—C10—H10B	108.3
C4—C3—H3A	119.9	O3—C11—O4	123.90 (15)
C2—C3—H3A	119.9	O3—C11—N2	124.31 (16)
C3—C4—C5	120.7 (2)	O4—C11—N2	111.78 (14)
C3—C4—H4A	119.6	C13—C12—O4	110.2 (2)
C5—C4—H4A	119.6	C13—C12—H12A	109.6
C4—C5—C6	118.7 (2)	O4—C12—H12A	109.6
C4—C5—H5A	120.7	C13—C12—H12C	109.6
C6—C5—H5A	120.7	O4—C12—H12C	109.6
C1—C6—C5	121.44 (18)	H12A—C12—H12C	108.1
C1—C6—S1	119.99 (14)	C12—C13—H13A	109.5
C5—C6—S1	118.55 (15)	C12—C13—H13B	109.5
N1—C7—C8	108.73 (12)	H13A—C13—H13B	109.5
N1—C7—H7A	109.9	C12—C13—H13C	109.5
C8—C7—H7A	109.9	H13A—C13—H13C	109.5
N1—C7—H7B	109.9	H13B—C13—H13C	109.5
			
O1—S1—N1—C10	175.82 (11)	N1—S1—C6—C5	85.65 (15)
O2—S1—N1—C10	46.71 (13)	C10—N1—C7—C8	−58.52 (17)
C6—S1—N1—C10	−68.69 (13)	S1—N1—C7—C8	163.26 (10)
O1—S1—N1—C7	−47.57 (12)	C11—N2—C8—C7	122.68 (18)
O2—S1—N1—C7	−176.67 (11)	C9—N2—C8—C7	−56.43 (18)
C6—S1—N1—C7	67.92 (12)	N1—C7—C8—N2	55.05 (16)
C6—C1—C2—C3	−0.5 (3)	C11—N2—C9—C10	−122.59 (17)
C1—C2—C3—C4	0.2 (4)	C8—N2—C9—C10	56.58 (19)
C2—C3—C4—C5	0.0 (4)	C7—N1—C10—C9	59.10 (17)
C3—C4—C5—C6	0.0 (3)	S1—N1—C10—C9	−162.49 (11)
C2—C1—C6—C5	0.5 (3)	N2—C9—C10—N1	−55.79 (18)
C2—C1—C6—S1	179.12 (15)	C12—O4—C11—O3	2.6 (3)
C4—C5—C6—C1	−0.3 (3)	C12—O4—C11—N2	−176.51 (17)
C4—C5—C6—S1	−178.91 (16)	C8—N2—C11—O3	178.53 (18)
O1—S1—C6—C1	21.89 (16)	C9—N2—C11—O3	−2.4 (3)
O2—S1—C6—C1	152.57 (14)	C8—N2—C11—O4	−2.4 (2)
N1—S1—C6—C1	−92.99 (15)	C9—N2—C11—O4	176.66 (15)
O1—S1—C6—C5	−159.47 (14)	C11—O4—C12—C13	−88.6 (3)
O2—S1—C6—C5	−28.79 (17)		

### Hydrogen-bond geometry (Å, °)

**Table:**

Cg1 is the centroid of the C1–C6 ring.

**Table:**

*D*—H···*A*	*D*—H	H···*A*	*D*···*A*	*D*—H···*A*
C13—H13A···Cg1^i^	0.96	2.97	3.900 (4)	165

Symmetry codes: (i) −*x*−1, −*y*, −*z*+1.
